# Serum Concentration of CRIPTO‐1 in Pregnancies Complicated by Placenta Accreta Spectrum

**DOI:** 10.1155/bmri/2476601

**Published:** 2026-07-09

**Authors:** Livia Alves Lopes, Mario Macoto Kondo, Estela Bevilacqua, Carolina Burgarelli Testa, Mario Henrique Burlacchini de Carvalho, Rossana Pulcineli Vieira Francisco, Fábio Roberto Cabar

**Affiliations:** ^1^ Hospital das Clínicas, University of São Paulo School of Medicine, São Paulo, Brazil, usp.br; ^2^ Instituto de Ciências Biomédicas, University of São Paulo, São Paulo, Brazil, usp.br

**Keywords:** antenatal prediction, biomarkers, CRIPTO-1, obstetric hemorrhage, placenta accreta spectrum, placenta previa

## Abstract

**Objective:**

To compare maternal serum CRIPTO‐1 concentrations in pregnancies complicated by placenta accreta spectrum (PAS) with those complicated by placenta previa (PP).

**Methods:**

We conducted a prospective, cross‐sectional, comparative study at a tertiary center in São Paulo, Brazil (August 2022–May 2025). Singleton pregnancies with ultrasound‐confirmed PP were included and classified as either PP without accreta or PAS (accreta/increta/percreta) according to standardized imaging criteria and, when applicable, surgical/histopathological confirmation. Maternal venous blood was collected predelivery, and serum CRIPTO‐1 concentrations were measured using ELISA (Human CRIPTO SimpleStep ELISA Kit, Abcam). Primary analyses used nonparametric tests for continuous variables and Fisher′s exact test for categorical variables. The study was approved by the Research Ethics Committee of the University of São Paulo (CAAE 58202122.8.0000.0065; CAAE 55121422.3.0000.0068).

**Results:**

Forty‐three participants were analyzed (PP = 18; PAS = 25). Median serum CRIPTO‐1 did not differ significantly between groups (PP: 0.013 pg/mL, IQR: 0.001–0.067 vs. PAS: 0.079 pg/mL, IQR: 0.005–0.360; *p* = 0.156). PAS was associated with greater blood loss (median 2240 vs. 568 mL; *p* < 0.001), higher transfusion rate (88.0% vs. 16.7%; *p* < 0.001), more ICU admissions (68.0% vs. 16.7%; *p* = 0.002), longer ICU and hospital stays (both *p* < 0.01), and higher hysterectomy rates (84.0% vs. 5.6%; *p* < 0.001).

**Conclusions:**

Predelivery serum CRIPTO‐1 did not discriminate PAS from PP, possibly reflecting physiological variability at delivery.

## 1. Introduction

Placenta accreta spectrum (PAS) represents one of the most complex challenges in modern obstetrics. Characterized by abnormal trophoblastic invasion of the uterine wall, PAS encompasses a continuum ranging from placenta accreta, in which villi attach directly to the myometrium, to increta and percreta, where invasion penetrates deeper into or beyond the uterine serosa. The clinical significance of PAS lies in its association with catastrophic hemorrhage, high rates of peripartum hysterectomy, and increased maternal–fetal morbidity and mortality. Over the past 3 decades, its incidence has increased in parallel with the global rise in cesarean deliveries and uterine instrumentation [1–4]. Current estimates suggest that PAS occurs in approximately 1 in every 500 pregnancies, although regional variation is wide and may be underestimated in low‐resource settings [5, 6].

Prenatal diagnosis of PAS is essential for planning delivery at tertiary centers, assembling multidisciplinary surgical teams, and minimizing hemorrhagic complications [7–10]. Ultrasound and magnetic resonance imaging (MRI) are currently the cornerstones of diagnosis, yet they rely heavily on operator expertise, availability, and equipment quality [8, 9]. In posterior or central placenta previa (PP), or in cases with excessive maternal adiposity or scarring, visualization of the placenta–myometrium interface can be suboptimal [10]. These limitations highlight the urgent need for complementary diagnostic approaches that could enhance early detection and risk stratification [11]. Several circulating biomarkers have been investigated as potential adjuncts to imaging in PAS, including alpha‐fetoprotein (AFP), pregnancy‐associated plasma protein‐A (PAPP‐A), human chorionic gonadotropin (*β*‐hCG), placental growth factor (PlGF), soluble fms‐like tyrosine kinase‐1 (sFlt‐1), and cell‐free fetal DNA, among others. Although some have shown associations with abnormal placentation, none has achieved sufficient sensitivity and specificity for routine clinical use, and important evidence gaps remain regarding optimal gestational timing and biomarker thresholds [12–14]. Against this background, CRIPTO‐1 represents a biologically plausible candidate for predicting PAS.

CRIPTO‐1 is a glycosyl‐phosphatidylinositol–anchored protein belonging to the epidermal growth factor–Cripto/FRL‐1/Cryptic (EGF‐CFC) family [15, 16]. It acts as a coreceptor in the Nodal signaling pathway and modulates key processes such as cell proliferation, migration, and epithelial–mesenchymal transition (EMT) [17]. In physiological pregnancy, these mechanisms facilitate trophoblastic invasion and spiral artery remodeling; however, their dysregulation may contribute to the excessive invasion observed in PAS [18–20]. Immunohistochemical studies have demonstrated overexpression of CRIPTO‐1 in villous and extravillous trophoblasts of accreta placentas, suggesting a possible link between CRIPTO‐1 signaling and abnormal placentation [21, 22].

Despite this biological plausibility, evidence regarding maternal serum concentrations of CRIPTO‐1 remains scarce. Circulating levels could potentially reflect placental activity and serve as a minimally invasive biomarker, but prior studies have been limited by small sample sizes and heterogeneity in gestational timing of sampling [23]. Most have focused on placental tissue expression rather than systemic detection [21–24]. Understanding whether CRIPTO‐1 is detectable and differentially expressed in maternal serum could pave the way for novel diagnostic or prognostic applications.

The present study was designed to compare serum CRIPTO‐1 concentrations in pregnancies complicated by PAS and in women with PP without accreta. We hypothesized that CRIPTO‐1 levels would be elevated in PAS due to enhanced trophoblastic invasiveness. In doing so, we sought to inform the development of biochemical markers that could complement imaging in the antenatal identification of PAS [11–14].

## 2. Materials and Methods

### 2.1. Study Design and Setting

This was a prospective, cross‐sectional comparative study conducted between August 2022 and May 2025 at a tertiary referral center in São Paulo, Brazil: the Department of Obstetrics and Gynecology of the University of São Paulo School of Medicine (Hospital das Clínicas–HCFMUSP). The institution is a national referral center for high‐risk obstetrics, offering advanced imaging, interventional radiology, and multidisciplinary management of PAS disorders.

The study followed the principles of the Declaration of Helsinki and adhered to STROBE (Strengthening the Reporting of Observational Studies in Epidemiology) recommendations for observational studies. Ethical approval was obtained from the institutional review boards of the hospital. Written informed consent was obtained from all participants prior to enrollment.

### 2.2. Participants and Classification Criteria

Eligible participants were women aged ≥ 18 years with singleton pregnancies, with no fetal malformations, and ultrasound‐confirmed PP diagnosed after 20 weeks gestation. Exclusion criteria included fetal demise, systemic infection or sepsis, failure to obtain a predelivery serum sample, or invalid CRIPTO‐1 assay results.

Participants were categorized into two groups based on antenatal imaging and intraoperative findings:

1. PP without evidence of abnormal invasion, and
2. PAS, comprising accreta, increta, and percreta subtypes.

The classification followed FIGO (2019) criteria and the standardized sonographic descriptors [9], including placental lacunae, loss of the clear zone, thinning or interruption of the uterine–bladder interface, and abnormal subplacental vascularity. Diagnosis was confirmed surgically and/or histopathologically when feasible. Of the 25 PAS cases, 21 were confirmed histopathologically, and four were classified based on intraoperative findings alone. All 18 PP cases were confirmed to have no histopathologic evidence of myometrial invasion. Women with PP without accreta were selected as the comparison group to control for placental location and focus the analysis on abnormal placental invasion.

### 2.3. Sample Collection and Processing

To minimize peridelivery physiological variation, maternal venous blood (5 mL) was drawn prior to cesarean delivery, before induction of anesthesia. Samples were collected in serum separator tubes and processed within 30 min. After centrifugation at 3000 rpm for 10 min, serum aliquots were stored at −80°C until analysis.

### 2.4. CRIPTO‐1 Quantification

Quantification of serum CRIPTO‐1 was performed using the Human CRIPTO SimpleStep ELISA Kit (ab245719, Abcam, Cambridge, United Kingdom) in accordance with the manufacturer′s protocol. Standards and samples were incubated for 2 h at 37°C in precoated microplates containing a monoclonal anti–CRIPTO‐1 antibody. After washing, a horseradish peroxidase–conjugated detection antibody was added, followed by tetramethylbenzidine (TMB) substrate. The reaction was stopped with 2 N sulfuric acid, and optical density was measured at 450 nm using a Bio‐Rad 680 microplate spectrophotometer.

Each sample was assayed in duplicate, and mean absorbance values were interpolated from a standard curve to yield results in pg/mL. Interassay and intra‐assay coefficients of variation below 10% were considered acceptable.

The Human CRIPTO SimpleStep ELISA Kit (ab245719) has a reported sensitivity of 9.6 pg/mL, with an intra‐assay coefficient of variation of 1.7% (*n* = 8) and an average serum‐specific recovery of 86%. Values below the sensitivity threshold of 9.6 pg/mL were recorded as detected but below the limit of quantification and are reported descriptively. Given that the majority of samples in both groups fell below this threshold, with group medians of 0.013 (PP) and 0.079 pg/mL (PAS), results should be interpreted with caution, as quantitative precision at these concentrations cannot be guaranteed by the manufacturer′s specifications.

### 2.5. Clinical and Perinatal Outcomes

Demographic, obstetric, and clinical data were extracted from electronic medical records. Maternal outcomes included estimated blood loss, need for blood transfusion, intensive care unit (ICU) admission, ICU length of stay, total hospitalization time, and hysterectomy. Neonatal outcomes included gestational age at delivery, birthweight, Apgar scores, prematurity (< 36 weeks), and neonatal intensive care unit (NICU) admission and duration.

## 3. Statistical Analysis

Data analysis was performed using IBM SPSS Statistics Version 25.0 (IBM Corp., Armonk, New York, United States). Distribution of continuous variables was evaluated using the Shapiro–Wilk and Kolmogorov–Smirnov tests. Normally distributed variables were expressed as mean ± standard deviation (SD) and compared using Student′s *t* test. Nonnormal variables were presented as median (interquartile range [IQR]) and compared using the Mann–Whitney U test. Categorical variables were analyzed using Fisher′s exact or the chi‐square tests, as appropriate. A two‐tailed *p* < 0.05 was considered statistically significant.

## 4. Results

### 4.1. Participant Characteristics

During the study period, 51 pregnant women with ultrasound‐confirmed PP, with or without PAS, were initially assessed for eligibility. After application of the inclusion and exclusion criteria, 43 participants were included in the final analysis: 25 (58.1%) in the PAS group and 18 (41.9%) in the PP without accreta group. Two patients were excluded because of fetal malformations, two because of twin pregnancies, three because of errors in CRIPTO‐1 assay measurements, and one because of an emergency delivery that precluded predelivery blood sample collection. Baseline characteristics are summarized in Table 1. The groups were comparable in maternal age (PP: 33 ± 5 years vs. PAS: 35 ± 5 years, *p* = 0.091). However, women with PAS had a significantly higher number of pregnancies and previous cesarean deliveries (*p* = 0.008 and *p* < 0.001, respectively), consistent with prior epidemiologic trends reported in the literature [3, 6, 11]. There were no significant differences in maternal comorbidities.

**Table 1:** Maternal demographic, clinical characteristics, and maternal outcomes.

Variable	Placenta previa (*n* = 18)	Placenta accreta spectrum (*n* = 25)	*p*
Maternal age (years), mean ± SD	33 ± 5	35 ± 5	0.091∗
Number of pregnancies, median (IQR)	3 (2–4)	4 (3–6)	0.008∗∗
Parity, *n* (%)			0.066∗∗∗
• Primiparous	3 (16.7%)	0 (0.0%)	
• Multiparous	15 (83.3%)	25 (100.0%)	
Previous abortions, median (IQR)	0 (0–1)	0 (0–1)	0.559∗∗
Previous cesarean deliveries, median (IQR)	1 (0–2)	2 (2–3)	< 0.001∗∗
Previous vaginal births, median (IQR)	0 (0–1)	0 (0–1)	0.726∗∗
CRIPTO‐1 (pg/mL), median (IQR)	0.013 (0.001–0.067)	0.079 (0.005–0.360)	0.156∗∗
Estimated blood loss (mL), median (IQR)	568 (494–834)	2240 (1860–4490)	< 0.001∗∗
Blood transfusion, *n* (%)	3 (16.7%)	22 (88.0%)	< 0.001∗∗∗
Maternal ICU admission, *n* (%)	3 (16.7%)	17 (68.0%)	0.002∗∗∗
ICU length of stay (days), median (IQR)	0 (0–0)	2 (0–3)	< 0.001∗∗
Hospital length of stay (days), median (IQR)	3 (2–4)	6 (3–10)	0.003∗∗
Peripartum hysterectomy, *n* (%)	1 (5.6%)	21 (84.0%)	< 0.001∗∗∗
Diabetes, *n* (%)	2 (11.1%)	7 (28.0%)	0.263∗∗∗
Hypertension, *n* (%)	0 (0.0%)	2 (8.0%)	0.502∗∗∗
Preeclampsia, *n* (%)	1 (5.6%)	1 (4.0%)	0.999∗∗∗
Thyroid disease, *n* (%)	3 (16.7%)	4 (16.0%)	0.999∗∗∗
Uterine fibroids, *n* (%)	0 (0.0%)	2 (8.0%)	0.502∗∗∗
Rheumatologic disease, *n* (%)	1 (5.6%)	1 (4.0%)	0.999∗∗∗
Mode of delivery, *n* (%)			0.717∗∗∗
• Elective cesarean	13 (72.2%)	20 (80.0%)	
• Emergency cesarean	5 (27.8%)	5 (20.0%)	

*Note:* Single asterisk “∗” denotes Student′s *t* test. Double asterisks “∗∗” denote Mann–Whitney U test. Triple asterisks “∗∗∗” denote Fisher′s exact test.

Abbreviations: IQR, interquartile range (1st–3rd quartile); SD, standard deviation.

### 4.2. Serum CRIPTO‐1 Concentrations

**Figure 1:**
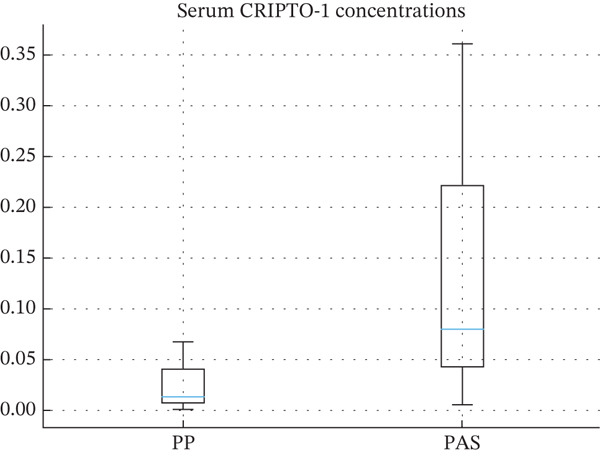
Serum CRIPTO‐1 concentrations in placenta previa and placenta accreta spectrum (PAS). Box‐and‐whisker plot showing serum CRIPTO‐1 concentrations (pg/mL) in pregnancies with placenta previa and PAS. The horizontal line represents the median, the box indicates the interquartile range, and dots represent individual values. No statistically significant difference was observed between groups (*p* = 0.156).

Median maternal serum CRIPTO‐1 concentrations did not differ significantly between the PP and PAS groups (PP = 0.013 pg/mL [IQR: 0.001–0.067] vs. PAS = 0.079 pg/mL [IQR: 0.005–0.360]; *p* = 0.156) (Figure 1). Although a wider range of values was observed in the PAS group, there was substantial overlap between distributions.

### 4.3. Maternal Outcomes

As expected, women with PAS experienced significantly higher maternal morbidity (Table 1). Estimated blood loss was markedly greater in PAS (median: 2240 mL [IQR: 1860–4490]) compared with PP (568 mL [IQR: 494–834]; *p* < 0.001), consistent with prior reports of massive hemorrhage in this population [1, 2, 4]. Blood transfusion was required in 88.0% of PAS versus 16.7% of PP cases (*p* < 0.001). ICU admission occurred in 68.0% versus 16.7% (*p* = 0.002). Median ICU stay was longer in PAS (2 days [IQR: 0–3]) than in PP (0 days [IQR: 0–0]; *p* < 0.001), as was hospital stay (6 days [IQR: 3–10] vs. 3 days [IQR: 2–4]; *p* = 0.003). Peripartum hysterectomy was required in 84.0% of PAS and 5.6% of PP patients (*p* < 0.001), consistent with the well‐documented severe maternal impact of PAS [2, 4, 14]. Intraoperative findings confirmed myometrial invasion in all PAS cases and bladder involvement in four (16%). There were no maternal deaths.

### 4.4. Neonatal Outcomes

Neonatal outcomes are summarized in Table 2. There were no statistically significant differences between groups in gestational age at delivery, prematurity, birthweight, Apgar scores, NICU admission, or NICU length of stay. Prematurity (< 36 weeks) occurred in 83.3% of the PP group and 92.0% of the PAS group (*p* = 0.634), consistent with the known contribution of placental pathophysiology to adverse perinatal outcomes [6, 10, 25].

**Table 2:** Neonatal outcomes.

Outcome	Placenta previa (*n* = 18)	Placenta accreta spectrum (*n* = 25)	*p*
Gestational age at delivery (weeks), mean ± SD	35.37 ± 1.53	34.89 ± 2.63	0.491∗
Prematurity (< 36 weeks), *n* (%)	15 (83.3%)	23 (92.0%)	0.634∗∗∗
Birthweight (g), mean ± SD	2537 ± 474	2519 ± 567	0.915∗
Apgar 1 min, median (IQR)	8 (7–8)	8 (7–8)	0.282∗∗
Apgar 5 min, median (IQR)	9 (8–9)	9 (8–9)	0.178∗∗
NICU admission, n (%)	9 (50.0%)	16 (64.0%)	0.532∗∗∗
NICU length of stay, *n* (%)			0.578∗∗∗
• No NICU days	9 (50.0%)	9 (36.0%)	
• 1–10 days	3 (16.7%)	7 (28.0%)	
• > 10 days	6 (33.3%)	9 (36.0%)	

*Note:* Single asterisk “∗” denotes Student′s t test. Double asterisks “∗∗” denote Mann–Whitney U test. Triple asterisks “∗∗∗” denote Fisher′s exact test.

Abbreviations: IQR, interquartile range (1st–3rd quartile); SD, standard deviation.

## 5. Discussion

This study investigated maternal serum CRIPTO‐1 concentrations in pregnancies complicated by PAS and compared them with cases of PP. Although circulating CRIPTO‐1 levels did not differ significantly between groups, the directional trend and its biological plausibility suggest an avenue worthy of further investigation. Our findings contribute to a growing body of research examining molecular pathways of abnormal placentation and emphasize the complexity of translating tissue‐level alterations into measurable circulating biomarkers.[5, 6, 11–14, 23].

CRIPTO‐1, also known as TDGF1, plays a fundamental role in embryogenesis and trophoblastic development. Acting as a coreceptor in the Nodal–SMAD2/3 signaling pathway, it mediates cellular differentiation, proliferation, and migration—processes that are essential for normal implantation and placental development [15–17, 26]. Studies have also indicated that CRIPTO‐1 plays fundamental roles introphoblastic EMT, a critical step enabling cytotrophoblasts to invade the maternal decidua and remodel uterine arteries [26, 27]. When CRIPTO‐1 signaling becomes dysregulated, trophoblasts may exhibit uncontrolled invasion similar to neoplastic behavior [21, 25, 28, 29]. This mechanistic overlap between cancer biology and PAS has been increasingly recognized, positioning CRIPTO‐1 as both a biological marker and a potential molecular driver of invasive placentation.

Histopathologic studies have demonstrated markedly increased CRIPTO‐1 expression in trophoblastic tissue from accreta and percreta cases compared with normal placentas [21, 28, 29]. Such upregulation may result from aberrant activation of the Nodal and Notch pathways, which together influence angiogenesis, extracellular matrix degradation, and the invasive phenotype of trophoblastic cells [21, 23, 26–29]. Nonetheless, the extent to which these tissue‐level differences translate into detectable serum changes remains uncertain. Circulating CRIPTO‐1 is subject to dilutional and metabolic effects, and peripartum factors such as hemorrhage, anesthesia, and surgical stress can further alter its concentration [23]. This biological variability may account for the absence of statistically significant intergroup differences in the present study, despite a consistent directional trend.

Our study supports the hypothesis that the timing of measurement is a crucial determinant of biomarker detectability. Sampling immediately prior to delivery, when extensive placental separation, uterine contraction, and hemodynamic instability occur, may obscure subtle intergroup differences [23]. Future longitudinal studies should therefore focus on serial antenatal measurements, ideally between 20 and 34 weeks of gestation, to evaluate whether CRIPTO‐1 trajectories diverge before the clinical manifestation of PAS [19, 21]. Combining such temporal profiling with Doppler velocimetry or MRI‐based placental perfusion imaging could provide a multidimensional understanding of disease progression [8–10].

Our study supports the hypothesis that the timing of measurement is a crucial determinant of biomarker detectability. Sampling immediately prior to delivery, when extensive placental separation, uterine contraction, and hemodynamic instability occur, may obscure subtle intergroup differences [23]. Future longitudinal studies should therefore focus on serial antenatal measurements, ideally between 20 and 34 weeks of gestation, to evaluate whether CRIPTO‐1 trajectories diverge before the clinical manifestation of PAS. Combining such temporal profiling with Doppler velocimetry or MRI‐based placental perfusion imaging could provide a multidimensional understanding of disease progression [8–10].

Although no statistically significant difference was observed, the trend toward higher median CRIPTO‐1 concentrations in PAS, together with prior histopathologic evidence of increased placental expression, supports a biologically plausible role for CRIPTO‐1 in abnormal placentation. The absence of significance in serum measurements may reflect the timing of sampling in the immediate predelivery period, when hemodynamic, inflammatory, and metabolic changes may attenuate subtle intergroup differences.

From a translational perspective, integrating biochemical markers such as CRIPTO‐1 into multimodal risk‐stratification algorithms could enhance prenatal triage. In resource‐limited settings, where expert sonography and MRI are scarce, a reliable serum biomarker panel could guide referrals to tertiary centers and improve maternal outcomes [1, 2, 9, 11, 23]. Advances in machine‐learning approaches may eventually allow personalized risk prediction through the integration of clinical data, obstetric history, imaging findings, and biomarker profiles [30]. In this context, CRIPTO‐1 might serve as one node within a broader predictive framework rather than a standalone test.

In the current literature, evidence on maternal serum CRIPTO‐1 in PAS is extremely limited. To our knowledge, Özköse et al. [23] remain the only prior study to quantify circulating CRIPTO‐1 in PAS, reporting higher antenatal serum concentrations in PAS cases when samples were obtained between 28 and 34 weeks under relatively stable conditions. In contrast, our study did not demonstrate a statistically significant difference between PAS and PP when CRIPTO‐1 was measured immediately before delivery (*p* = 0.156). This discrepancy is plausibly explained by timing and methodological differences: Antenatal sampling may better capture placental signaling in a less perturbed physiologic state, whereas predelivery measurements are influenced by hemodilution, acute inflammatory and metabolic shifts, hemorrhage, and therapeutic interventions, which may obscure subtle intergroup differences in circulating concentrations. These findings underscore the importance of future studies evaluating longitudinal antenatal trajectories of CRIPTO‐1 and harmonized sampling protocols to clarify its diagnostic and/or prognostic utility in PAS.

The current study has several limitations. The sample size was modest, limiting statistical power to detect subtle differences. Measurements were restricted to a single predelivery time point, precluding assessment of longitudinal variation. Furthermore, the observed serum concentrations in a subset of participants, particularly in the PP group, approached or fell below the manufacturer′s reported lower limit of detection for the assay, which may have introduced measurement uncertainty at the lower end of the distribution and should be interpreted with caution. Additionally, we measured total serum CRIPTO‐1 without differentiating between isoforms and posttranslational modifications that may carry distinct biological functions [24]. Nevertheless, our study′s strengths include a prospectively collected cohort, standardized ELISA methodology, and rigorous diagnostic confirmation of PAS using surgical and histopathologic correlation.

### 5.1. Future Directions

Further research should be aimed at (1) determining gestational profiles of CRIPTO‐1 and related markers such as Nodal, activin A, and soluble VEGF receptor; (2) exploring molecular interactions within the TGF‐*β* superfamily that drive aberrant trophoblastic invasion; and (3) evaluating the performance of CRIPTO‐1 in combination with established imaging and clinical predictors. Multicenter prospective studies using harmonized protocols would facilitate validation and meta‐analytic synthesis of findings.

## 6. Conclusion

Predelivery serum CRIPTO‐1 concentrations did not significantly differ between pregnancies with PAS and those with uncomplicated PP. Nevertheless, the observed trend toward higher levels in PAS suggests a potential link between CRIPTO‐1 and the pathophysiology of invasive placentation.

This study highlights both the promise and the challenges of translating molecular discoveries into clinical practice. CRIPTO‐1 may not yet serve as a stand‐alone diagnostic biomarker, but its integration with imaging, clinical, and computational approaches could advance early identification of high‐risk patients and inform individualized obstetric management strategies [1, 4, 6–9]. Continued investigation of CRIPTO‐1 signaling pathways may ultimately support the development of personalized, biomarker‐guided obstetric care for women with PAS disorders.

## Funding

This study was supported by Universidade de São Paulo (10.13039/501100005639).

## Conflicts of Interest

The authors declare no conflicts of interest.

## Data Availability

The data supporting this study are not publicly available due to institutional and patient privacy restrictions but may be obtained from the corresponding author upon reasonable request and with permission from Hospital das Clínicas–HCFMUSP and Fleury S.A.
